# Examining the Magnitude of Maternal Ethnic and Socioeconomic Inequalities on Foetal Growth Restriction and Preterm Birth: A Cohort Study Set in North West England

**DOI:** 10.1007/s40615-025-02437-2

**Published:** 2025-04-16

**Authors:** Omowunmi Omole, Victoria Palin, Kylie Watson, Jenny Myers

**Affiliations:** 1 Maternal and Fetal Research Centre, Division of Developmental Biology and Medicine, the University of Manchester, Oxford Road, Manchester, M13 9 WL UK; 2 St Mary’s Hospital, Manchester Foundation Trust, Manchester, M13 9 WL UK

**Keywords:** Adverse pregnancy outcome,, Health inequalities,, Social deprivation,, Ethnic minorities,, Foetal growth restriction,, Preterm birth

## Abstract

**Objective:**

To calculate population attributable fractions (PAFs) for the effect of ethnicity and deprivation on foetal growth restriction (FGR) and preterm birth (PTB). PAF estimates the risk reduction of FGR and PTB if ethnic and socioeconomic inequalities did not exist.

**Design:**

A retrospective cohort study using routinely recorded electronic health records, 2016–2021, Manchester, UK.

**Methods:**

Women with singleton pregnancies greater than 22 weeks’ gestation. Logistic regression models were fitted to explore the association between maternal self-reported ethnicity, or deprivation (index of multiple deprivation) on the odds of developing foetal growth restriction (FGR) and preterm birth (PTB). PAFs were estimated from (un)adjusted logistic regression models.

Main Outcome Measures.

The PAF of FGR and PTB cases associated with ethnicity and deprivation.

**Results:**

A total of 48,930 pregnancies were included in the analysis with FGR and PTB rates of 8.5% and 6.9%, respectively. Forty-five percent were from ethnic minority backgrounds with 33% living in the most deprived postcode wards. In adjusted models, 22.8% (95% CI 19.6–25.9%) of FGR cases were attributable to ethnicity (using White British/Irish as comparison group). There was no effect of ethnicity on the PAF of PTB cases. In comparison to women living in the least deprived tertile of our population, 9.1% (95% CI 4.6–13.5%) of FGR cases and 11.2% (95% CI 6.2–15.9%) of PTB cases were attributable to deprivation.

**Conclusions:**

In our population, there is a disparity in pregnancy outcomes for women of ethnic minorities and those living in deprived areas. Targeted interventions such as antenatal caseload models and improved screening in high-risk women could contribute to the efforts to reduce maternal and perinatal morbidity in the UK.

**Supplementary Information:**

The online version contains supplementary material available at 10.1007/s40615-025-02437-2.

## Introduction

Reducing the incidence of perinatal mortality remains a priority in maternal and foetal health worldwide. In the UK, the implementation of guidelines such as Better Births [1] and Saving Babies Lives [2] has helped to reduce perinatal mortality. However, inequalities persist. Key drivers of these inequalities might be a result of ethnic and socioeconomic inequalities established in foetal growth restriction (FGR) and preterm birth (PTB), major risk factors of perinatal mortality [3].

Foetal growth restriction (FGR) at birth is defined as birthweight less than the third centile or below the 10 th centile if birth is < 34 weeks [4]. The aetiology of FGR is multifactorial, with maternal, foetal, and placental factors implicated [5]. FGR is associated with an increased ongoing risk of cardiovascular, respiratory, and neurological morbidity and mortality in early and later life [6]. According to the World Health Organisation (WHO), preterm birth (PTB) is defined as birth before 37 completed weeks’ gestation. Increased incidence of iatrogenic deliveries for FGR is also driving the prevalence of PTB [7]. Similarly, PTB is associated with an increased risk of morbidity and mortality [8].

A population study conducted in London showed that all ethnic minority women had an increased risk of having small-for-gestational-age (SGA) babies compared to White British women [9]. Black women (OR 2.35) and South Asian (OR 2.29) had the highest risk of having SGA babies. The population attributable fraction (PAF) is defined as the proportion of cases of an adverse outcome attributed to an exposure [10]. A national cohort study conducted in England (2015–17) showed that 19.5% of FGR cases were attributable to ethnicity, where the biggest effect of ethnicity and deprivation was seen in South Asian women [3]. In the same study, it was found that ethnicity had no overall effect on the risk of PTB. However, for minoritised ethnic women living in the most deprived areas, there was an increased risk of PTB. An older (2006–2012) retrospective cohort study conducted in England by Li et al. [7] also showed that all ethnic minority groups had an increased risk of PTB in comparison to White British women. Further, Li et al. concluded that the higher infant mortality rate in Black groups was a result of complications from extremely preterm births [7].

Deprivation has also previously been shown to be associated with adverse pregnancy outcomes. A population study conducted across England and Wales (2006–2012) showed that women living in the most deprived areas had an increased risk of PTB in comparison to women living in the least deprived areas (OR 1.49) [11]. Further, the study concluded that 78.2% of PTB cases in Black African women were mediated by their socioeconomic circumstances. Hirst et al. [12] also found that women living in the most deprived areas (2011–2012) had an increased risk of having an SGA neonate compared to women living in the least deprived areas.

The aim of the current study was to determine the effect of ethnicity and deprivation in a tertiary hospital catering to an ethnically dense and deprived population in North-West England so that health inequalities within our local population could be tackled. Our aim was to determine the disparities in rates of FGR and PTB, following adjustment for important confounders, such as smoking and common pre-pregnancy medical conditions. Our sample size also enabled the effect of ethnicity to be estimated in some individual ethnic groups as opposed to the aggregated ethnic groups which have been used in previous studies. Although ethnicity and deprivation are non-modifiable factors from a healthcare perspective, the identification of specific groups within a hospital locality has the potential to enable targeted interventions at a community-based level.

## Method

### Study Population

Health Research Authority approval was granted for the use of routine data (21/HRA/2377). We included all health records for women aged 16–55 who delivered a singleton pregnancy at St Mary’s Hospital, Oxford Road Campus (ORC), Manchester, UK, between January 2016 and December 2021. All records were anonymised prior to the analysis after extraction from the routine electronic health records (Ciconia Maternity Information System (CMiS)). Information regarding maternal demographics (self-reported ethnicity, postcode at booking, age, height, weight, history of diabetes and hypertension, smoking status and parity) and pregnancy outcomes (onset of labour, birth gestation, infant sex, birthweight, mode of birth, estimated blood loss) were included. Pregnancies complicated by a congenital anomaly or where the birth occurred before 22 weeks' gestation were excluded from the analysis.

### Exposures

Maternal ethnicity was self-reported at the booking visit according to the 2001 UK census categories. Index of multiple deprivation (IMD) deciles were derived from maternal booking postcodes. IMD deciles were calculated by ranking and dividing lower super output areas (LSOAs) into 10 equal groups from most deprived to least deprived. To allow meaningful analysis of deprivation, IMD deciles were grouped into three categories, each representing around one-third of our population (Supplementary Fig. 1). Women living in areas in decile 1 were assigned to the most deprived group, areas in IMD deciles 2 to 3 the middle deprivation group, and IMD deciles 4 to 10 the least deprived group. Maternal characteristics associated with adverse pregnancy outcomes, including maternal age, body mass index (BMI), parity (nulliparous or multiparous), pre-existing hypertension, maternal diabetes and smoking status, were included in the adjusted models [2].

### Outcome Measures

Two primary outcomes were investigated: 1) FGR was defined as birthweight below the 3rd centile or below the 10 th centile before 34-week gestation using the WHO sex-adjusted population centiles [13], and 2) PTB was defined as delivery before 37 completed weeks’ gestation.

### Statistical Analysis

Normally distributed and non-normally distributed continuous variables were compared using the ANOVA and Kruskal–Wallis test, respectively. The prevalence of each outcome was compared between groups using Pearson’s chi-squared test. The population attributable fraction (PAF) for the effect of ethnicity and deprivation was calculated from mixed logistic regression models (random effects women with repeat singleton pregnancies in the dataset), estimating the effect for binary outcome FGR or PTB separately with and without adjustment for confounding variables. Population PAFs were calculated by comparing the whole population with the reference group. Group-specific PAFs for ethnic groups stratified by area deprivation were calculated by comparing specific groups with the reference group.

PAFs described the proportion of FGR or PTB cases in each group that could be attributed to differences in ethnicity and/or deprivation (calculated as the percentage of excess cases compared to a theoretical dataset where the prevalence of cases is estimated from the reference group (i.e., White British/Irish ethnicity, least-deprived group).

As the analysis included an opportunistic sample (births recorded over a 5-year period), a formal a priori sample size calculation was not performed. For the smallest ethnic group (*n* = 272, Other Asian), the available sample had ≥ 80% power to detect an OR of ≥ 1.75. In the larger ethnic minority groupings, the sample provided sufficient power to detect ORs of ≥ 1.3–1.4. All analyses were conducted using Stata version 17.

## Results

**Fig. 1:**
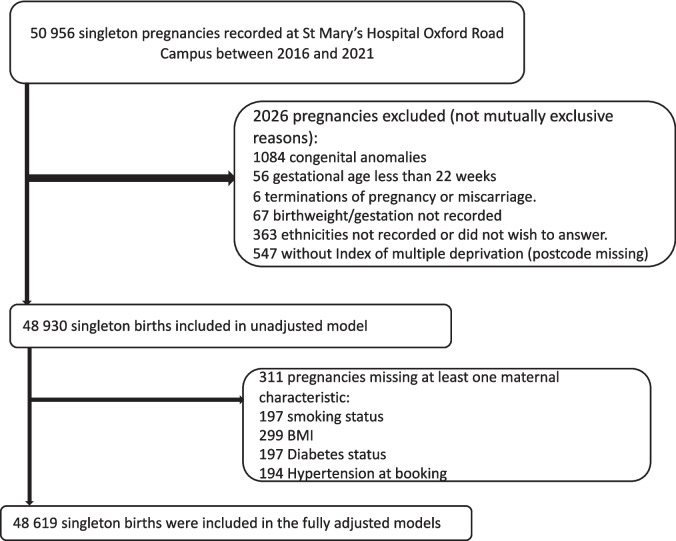
Inclusion of pregnancies in the study analysis

There were 50,956 singleton births at St Mary’s Hospital Oxford Road Campus between 2016 and 2021 (Fig. 1). 1,084 (2.13%) of pregnancies were complicated by congenital anomalies, 62 (0.12%) pregnancies ended in termination or a miscarriage prior to 22 weeks and in 363 (0.71%) ethnicity, 547 (1.1%) postcode and 67 (0.13%) birthweights were not recorded. Following exclusions, 48,930 pregnancies (from 40,120 women) were included in the analysis; 48,619 pregnancies (from 39,874 women) had complete data sets and were included in the adjusted models (Fig. 1).

### Baseline Characteristics

Characteristics of the birth cohort are shown in Table 1. Fifty-four percent of women were from ethnic minority backgrounds, and 36% of women lived in the most deprived (decile) postcode wards. FGR and PTB complicated 8.5 and 6.9% of pregnancies, respectively. Poor outcomes were more common in nulliparous pregnancies. Iatrogenic birth was more common in pregnancies complicated by FGR and PTB.

**Table 1:** Baseline characteristics of the birth cohort, stratified by outcome

	**Total**	**FGR**	**PTB**	***p*-value**^**§**^
	*N* = 48,930	*N* = 4137 (8.5%)	*N* = 3400 (6.9%)	
**Deprivation group**				< 0.001
Least deprived	16,325 (33.4%)	1160 (28.0%)	1010 (29.7%)	
Middle deprivation	15,064 (30.8%)	1365 (33.0%)	1073 (31.6%)	
Most deprived	17,541 (35.8%)	1612 (39.0%)	1317 (38.7%)	
**Ethnicity**				< 0.001
White British/Irish	22,432 (45.8%)	1570 (38.0%)	1720 (50.6%)	
Black Caribbean	842 (1.7%)	109 (2.6%)	72 (2.1%)	
Black African	5198 (10.6%)	469 (11.3%)	361 (10.6%)	
Black mixed	996 (2.0%)	95 (2.3%)	68 (2.0%)	
Pakistani	6408 (13.1%)	790 (19.1%)	447 (13.1%)	
Indian	1374 (2.8%)	201 (4.9%)	93 (2.7%)	
Bangladeshi	923 (1.9%)	134 (3.2%)	62 (1.8%)	
Other Asian	1837 (3.8%)	145 (3.5%)	75 (2.2%)	
Chinese	800 (1.6%)	55 (1.3%)	47 (1.4%)	
Other Black	399 (0.8%)	34 (0.8%)	25 (0.7%)	
Other White	4416 (9.0%)	259 (6.3%)	234 (6.9%)	
Asian Mixed	272 (0.6%)	30 (0.7%)	15 (0.4%)	
Other	3033 (6.2%)	246 (5.9%)	181 (5.3%)	
**Maternal age**				< 0.001
18 and younger	218 (0.4%)	24 (0.6%)	20 (0.6%)	
18–25	7399 (15.1%)	795 (19.2%)	544 (16.0%)	
25– < 30	13,454 (27.5%)	1231 (29.8%)	880 (25.9%)	
30– < 35	15,828 (32.3%)	1177 (28.5%)	1050 (30.9%)	
35– < 40	9563 (19.5%)	725 (17.5%)	681 (20.0%)	
40 and older	2468 (5.0%)	185 (4.5%)	225 (6.6%)	
**BMI group**				< 0.001
< 18.5	1516 (3.1%)	264 (6.4%)	133 (3.9%)	
18.5–25	21,550 (44.0%)	2009 (48.6%)	1368 (40.2%)	
25– < 30	14,592 (29.8%)	1094 (26.4%)	942 (27.7%)	
30– < 35	6917 (14.1%)	483 (11.7%)	533 (15.7%)	
35– < 40	2690 (5.5%)	171 (4.1%)	229 (6.7%)	
≥ 40	1369 (2.8%)	65 (1.6%)	113 (3.3%)	
Missing	296 (0.6%)	51 (1.2%)	82 (2.4%)	
**Parity**				< 0.001
0	19,890 (40.6%)	2127 (51.4%)	1390 (40.9%)	
1	15,344 (31.4%)	1023 (24.7%)	921 (27.1%)	
2	7366 (15.1%)	498 (12.0%)	492 (14.5%)	
≥ 3	6274 (12.8%)	480 (11.6%)	573 (16.9%)	
Missing	56 (0.1%)	9 (0.2%)	24 (0.7%)	
**Smoker**	6759 (13.8%)	875 (21.2%)	664 (19.5%)	< 0.001
Missing	196 (0.4%)	38 (0.9%)	57 (1.7%)	
**Gestation at Booking (w)**				< 0.001
Less than 10	13,817 (28.2%)	1260 (30.5%)	1074 (31.6%)	
10–13	23,111 (47.2%)	1762 (42.6%)	1346 (39.6%)	
13–20	7073 (14.5%)	614 (14.8%)	495 (14.6%)	
≥ 20	4889 (10.0%)	498 (12.0%)	480 (14.1%)	
Missing	40 (0.1%)	3 (0.1%)	5 (0.1%)	
**Diabetes status at booking**				< 0.001
Previous GDM	1425 (2.9%)	65 (1.6%)	120 (3.5%)	
Type1	225 (0.5%)	12 (0.3%)	75 (2.2%)	
Type2	330 (0.7%)	20 (0.5%)	73 (2.1%)	
Missing	196 (0.4%)	36 (0.9%)	57 (1.7%)	
**Hypertension at booking**	1782 (3.6%)	194 (4.7%)	282 (8.3%)	< 0.001
Missing	193 (0.4%)	35 (0.8%)	56 (1.6%)	
**Perinatal death**				< 0.001
Neonatal death	37 (0.1%)	18 (0.4%)	34 (1.0%)	
Stillbirth	171 (0.3%)	87 (2.1%)	126 (3.7%)	
**Gestation at birth (days)**	277 (270–284)	271 (257–282)	248 (232–255)	< 0.001
**WHO birth centile**	31.0 (12.0–57.0)	1.0 (0.0–2.0)	23.0 (5.0–54.0)	< 0.001
**Centile group**				< 0.001
< 3rd	3685 (7.5%)	3685 (89.1%)	680 (20.0%)	
3rd– < 10th	6568 (13.4%)	452 (10.9%)	452 (13.3%)	
10th– < 90th	35,682 (72.9%)	0 (0.0%)	2,011 (59.1%)	
90th– < 97th	1847 (3.8%)	0 (0.0%)	115 (3.4%)	
≥ 97th	1148 (2.3%)	0 (0.0%)	142 (4.2%)	
**FGR**	4137 (8.5%)	4137 (100.0%)	1132 (33.3%)	< 0.001
**FGR (birth < 37 weeks)**	680 (1.4%)	680 (16.4%)	680 (20.0%)	< 0.001
**Preterm birth (< 37 weeks)**	3400 (6.9%)	1132 (27.4%)	3400 (100.0%)	< 0.001
**Preterm birth (< 34 weeks)**	1045 (2.1%)	401 (9.7%)	1045 (30.7%)	< 0.001
**Birth gestation group**				< 0.001
< 28 weeks	291 (0.6%)	98 (2.4%)	291 (8.6%)	
28– < 32 weeks	350 (0.7%)	155 (3.7%)	350 (10.3%)	
32– < 37 weeks	2759 (5.6%)	879 (21.2%)	2759 (81.1%)	
≥ 37 weeks	45,530 (93.1%)	3005 (72.6%)	0 (0.0%)	
**Onset of labour**				< 0.001
Induced	14,423 (29.5%)	1366 (33.0%)	708 (20.8%)	
No labour	12,840 (26.2%)	1382 (33.4%)	1562 (45.9%)	
Spontaneous	21,666 (44.3%)	1389 (33.6%)	1130 (33.2%)	
Missing	1 (0.0%)	0 (0.0%)	0 (0.0%)	
**Method of birth**				< 0.001
Forceps	5264 (10.8%)	352 (8.5%)	171 (5.0%)	
Ventouse	2035 (4.2%)	208 (5.0%)	50 (1.5%)	
Elective Caesarean	8019 (16.4%)	587 (14.2%)	699 (20.6%)	
Emergency Caesarean	6730 (13.8%)	873 (21.1%)	789 (23.2%)	
Spontaneous vaginal	26,874 (54.9%)	2117 (51.2%)	1691 (49.7%)	
Missing	8 (0.0%)	0 (0.0%)	0 (0.0%)	
**Post-partum haemorrhage**				< 0.001
No PPH (500 mls)	36,454 (74.5%)	3303 (79.8%)	2632 (77.4%)	
Minor PPH (500– < 1000 mls)	9210 (18.8%)	632 (15.3%)	521 (15.3%)	
Major PPH (≥ 1000 mls)	2840 (5.8%)	160 (3.9%)	217 (6.4%)	
Missing	426 (0.9%)	42 (1.0%)	30 (0.9%)	

*GDM*, gestational diabetes;

^§^*P*-value represents the difference between preterm birth vs no preterm birth and FGR vs no FGR for each categorical variable

Outputs from the unadjusted and adjusted logistic regression models are included in Supplementary Tables 1 and 2. Black African, Black Caribbean, Pakistani, Indian, and Bangladeshi ethnicity were associated with increased odds of FGR (odds ratios 1.4–2.0). In contrast, ethnicity was not associated with an increased risk of PTB in either unadjusted or adjusted models, with some ethnic minority groups (other Asian, other White) having a reduced risk. The risk of both FGR and PTB was consistently associated with deprivation in both adjusted and unadjusted models. As expected, smoking, maternal hypertension and diabetes history recorded at the time of booking were associated with significantly increased risks of both FGR and PTB. An increase in BMI was associated with a reduced risk of FGR and was not significantly associated with PTB.

### Population-Level Attributable Fractions

**Fig. 2:**
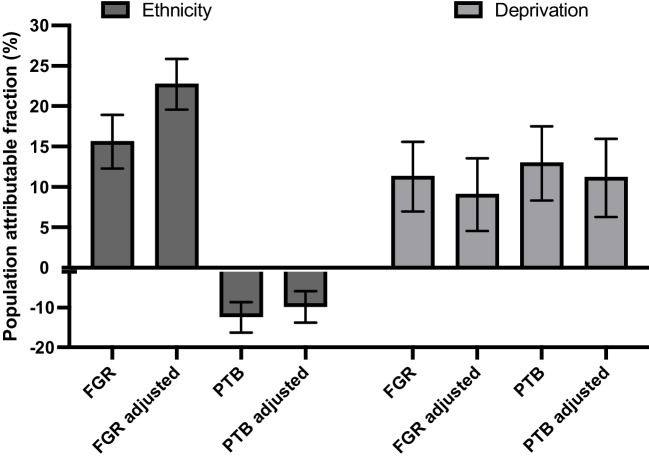
Population-specific population attributable fractions calculated for the effect of ethnicity and deprivation on FGR and PTB. The PAF is an estimation of the proportion of cases in each group attributable to the difference in ethnicity and deprivation (compared to the White British/Irish group in the least deprived group). Bars represent PAF with 95% CI calculated from logistic regression models including ethnicity and deprivation (FGR, PTB) and fully adjusted models including smoking, maternal age, BMI, maternal diabetes and hypertension

In the observed population, 15.7% (12.3–18.9%) of FGR cases were found to be attributable to ethnicity (using White British/Irish as the reference group) (Fig. 2). After adjustment for all available confounding variables, 22.8% (95% CI 19.6–25.9%) of cases were estimated to be attributable to differences in ethnicity. Ethnicity was not associated with an increase in PTB cases. Adjustment for confounders did not significantly affect the PAF associated with deprivation (Fig. 2). In the fully adjusted models, 9.1% (95% CI 4.6–13.5%) of FGR cases and 11.2% (95% CI 6.2–15.9%) of PTB were estimated to be attributable to the level of deprivation.

### Group-Specific Attributable Fractions

The group-specific PAF (stratified by ethnic group and deprivation tertile) are shown in Tables 2 and 3. The percentage of FGR cases associated with deprivation amongst White British/Irish Women living in the middle and most deprived areas in our locality was 12 and 14%, respectively (Table 2). The proportion of FGR cases attributable to ethnicity differences between the group and the reference population was highest in South Asian women: 59–64% in Indian women, 55–62% in Pakistani women and 61–66% in Bangladeshi women. The proportion of FGR cases attributable to the difference in ethnic background between the Black Caribbean group (50–56%) and the reference group was slightly higher than the difference between the Black African group (38–47%) and the reference group across deprivation groups.

**Table 2:** Group-specific population attributable fractions (95% CI) for FGR calculated from adjusted logistic regression models, stratified by ethnicity and deprivation

Ethnic group	Least deprived	Middle deprivation group	Most deprived
White British/Irish	Reference group	**11.7% (4.3–18.6%)** ***N*** = 6028	**14.4% (7.3–21.0%)** ***N*** = 6285
Black Caribbean	**49.8% (39.4–58.4%)** ***N*** = 184	**54.8% (45.1–62.7%) *N*** = 248	**56.5% (47.2–64.2%)** ***N*** = 409
Black African	**38.4% (31.2–44.8%)** ***N*** = 541	**45.6% (38.1–52.1%)** ***N*** = 1335	**47.4% (40.8–53.4%)** ***N*** = 3292
Black mixed	**21.6% (3.2–36.5%)** ***N*** = 228	**30.1% (13.3–43.7%)** ***N*** = 281	**32.2% (16.2–45.2%)** ***N*** = 483
Pakistani	**55.5% (51.3–59.3%)** ***N*** = 1475	**60.4% (55.8–64.5%)** ***N*** = 2752	**61.7% (57.2–65.8%)** ***N*** = 2153
Indian	**59.0% (52.6–64.6%)** ***N*** = 587	**63.3% (57.0–68.7%)** ***N*** = 506	**64.0% (57.9–69.3%)** ***N*** = 273
Bangladeshi	**60.9% (53.5–67.2%)** ***N*** = 126	**65.1% (58.2–70.9%)** ***N*** = 365	**66.0% (59.3–71.5%)** ***N*** = 430
Other Asian	**26.8% (13.1–38.4%)** ***N*** = 446	**35.8% (22.7–46.6%)** ***N*** = 648	**37.9% (25.4–48.3%)** ***N*** = 734
Asian mixed	**36.7% (9.8–55.6%)** ***N*** = 91	**43.5% (19.7–60.3%)** ***N*** = 88	**44.8% (22.0–60.9%)** ***N*** = 92
Other	**23.4% (12.2–33.1%)** ***N*** = 798	**32.3% (21.2–41.7%)** ***N*** = 991	**34.5% (23.9–43.6%)** ***N*** = 1218

The PAF is an estimation of the proportion of cases in each group attributable to the difference in ethnicity and deprivation (compared to the White British/Irish group in the least deprived group). Significant results are shown in bold

**Table 3:** Group-specific population attributable fractions (95% CI) for PTB calculated from adjusted logistic regression models, stratified by ethnicity and deprivation

Ethnic group	Least deprived	Middle deprivation group	Most deprived
White British/Irish	Reference group	**14.5% (6.8–21.6%)** ***N*** = 6028	**16.7% (9.3–23.6%)** ***N*** = 6285
Black Caribbean	5.9% (− 19.5 to 25.9%) *N* = 184	19.3% (− 2.9 to 36.8%) *N* = 248	**21.4% (0.1–38.1%)** ***N*** = 409
Black African	− 12.7% (− 27.6 to 0.5%) *N* = 541	4.0% (− 10.5 to 16.6%) *N* = 1335	3.8% (− 23.7 to 25.2%) *N* = 483
Black mixed	− 16.2% (− 49.0 to 9.4%) *N* = 228	1.0% (− 27.6 to 23.2%) *N* = 281	3.8% (− 23.7 to 25.2%) *N* = 483
Pakistani	− 6.8% (− 19.4 to 4.5%) *N* = 1475	8.9% (− 3.6 to 19.9%) *N* = 2752	11.4% (− 0.9 to 22.2%) *N* = 2153
Indian	− 5.6% (− 30.9 to 14.7%) *N* = 587	10.0% (− 12.4 to 28.0%) *N* = 506	12.5% (− 9.3 to 29.9%) *N* = 273
Bangladeshi	− 20.2% (− 56.5 to 7.6%) *N* = 126	− 2.3% (− 33.0 to 21.3%) *N* = 365	0.6% (− 28.9 to 23.3%) *N* = 430
Other Asian	− 82.2% (− 131.3 to − 43.6%) *N* = 446	− 54.5% (− 98.1 to − 20.5%) *N* = 648	− 49.7% (− 91.4 to − 17.1%) *N* = 734
Asian mixed	− 45.3% (− 144.8 to 13.7%) *N* = 91	− 23.3% (− 107.0 to 26.6%) *N* = 88	− 18.7% (− 94.1 to 27.4%) *N* = 92
Other	− 24.4% (− 45.8 to − 6.2%) *N* = 798	− 5.6% (− 25.7 to 11.2%) *N* = 991	− 2.6% (− 21.5 to 13.4%) *N* = 1218

The PAF is an estimation of the proportion of cases in each group attributable to the difference in ethnicity and deprivation (compared to the White British/Irish group in the least deprived group). Significant results are shown in bold

The PAFs for PTB did not identify many differences between ethnicity/deprivation groups (Table 3). In White British/Irish women living in the middle and most deprived areas, 15–17% of PTB cases were estimated to be attributable to deprivation. Compared to the reference group, 21% of PTB cases in Black Caribbean women living in the most deprived areas were attributable to ethnicity and deprivation.

Given the large variability in the aetiology of FGR and PTB, two sensitivity analyses were performed to determine whether there was significant variation in the association between ethnicity and deprivation in pregnancies with FGR where the birth occurred before 37 weeks (Supplementary Table 3) and PTB where the onset of labour was spontaneous (Supplementary Table 4). The magnitude and direction of the ORs were largely unchanged. Black African, Black Caribbean, Pakistani, Indian, and Bangladeshi ethnicity remained associated with preterm FGR, although the ORs were smaller; as expected, maternal hypertension was the strongest risk factor for preterm FGR (OR 4.25 [95% CI 3.04–5.94]). Black African, Pakistani, other Asian, and other White ethnic groups all had modestly reduced odds of spontaneous PTB; smoking (OR 2.1 [1.68–2.62]), deprivation (OR 1.36 [1.10–1.67]), and medical comorbidities continued to be associated with an increased risk. Outcomes stratified by each ethnicity group are shown in Supplementary Table 5.

## Discussion

In line with previous studies, our study, set in an urban, diverse population in the North West of England, has identified significant differences in the prevalence of FGR and PTB between women from different ethnic backgrounds living in different areas of deprivation. Following adjustment for confounders known to be associated with adverse pregnancy outcomes, we identified a number of ethnic groups where a high proportion (> 50%) of cases of FGR were estimated to be attributable to differences in ethnicity. A smaller (10–15%) proportion of FGR cases were attributable to deprivation. The proportion of FGR cases attributable to ethnicity was highest in all South Asian women and Black ethnic minority groups. Conversely, the risk of PTB was not found to be attributable to ethnicity, but 10–15% of PTB cases were estimated to be attributable to differences in deprivation.

### Interpretation

Several previous studies have demonstrated ethnic and socioeconomic inequalities in perinatal outcomes [3, 11, 12], and most studies have shown the highest excess risk of FGR, in terms of ethnicity and deprivation, in South Asian women living in the most deprived areas, in agreement with our study [3]. Other studies have evaluated the effect of ethnicity in aggregated ethnic groups [3, 11]. In our study, we were able to use some more specific ethnic groups and found that women from Bangladeshi backgrounds had the highest excess risk of FGR. Previous studies have reported conflicting findings in terms of variation in PTB rates by ethnicity [3, 7, 11, 14]; the largest most recent study in England [3] did not identify a big impact of ethnicity on PTB (PAF 1.2% (0.8–1.6), in agreement with findings from our local population.

In terms of the effect of deprivation, Opondo et al. [11] found that 19.3% (17–21.5%) of the risk of PTB was mediated by socioeconomic deprivation in Black Caribbean women and 78.2% (64.2–92.2%) of the effect of ethnicity on PTB was mediated by deprivation in Black African women. In our study, women with Black Caribbean ethnicity living in the most deprived areas had an excess risk of PTB. Deprivation was also associated with an increased risk amongst women of White British/Irish ethnicity in our population; findings are important to consider in terms of developing local strategies to improve outcomes.

Previous studies have included some confounders in their analysis. For example, in the national cohort study by Jardine et al. [3], the effect of ethnicity was reduced after adjustment for area deprivation, but disparities between minority ethnic groups were increased after adjustment for smoking and BMI. In our study, we were able to include additional important confounders, including maternal diabetes and hypertension reported at booking, in addition to smoking status, parity, maternal age and BMI. We also found an increase in the proportion of FGR cases attributable to ethnicity differences following adjustment for confounders. It is possible that underreporting maternal comorbidities at booking amongst different ethnic groups may explain this finding or that there is hidden confounding that has not been captured in our study. However, given the very significant proportion of FGR cases attributable to ethnicity differences, it is clear that several ethnic minority groups in our population have a very significantly increased risk of FGR; it is imperative that screening programmes are tailored to maximise the detection of FGR in these groups and that surveillance and intervention are targeted to high-risk pregnancies. Notably, risks among some ethnic minority groups remain high even in the least deprived strata, suggesting that ethnicity is not simply a proxy for deprivation in our population. This points to the potential role of broader structural determinants, including systemic inequalities, discrimination, differential access to care, and social exclusion. Future research is needed to disentangle these overlapping influences and to better understand how ethnic and socioeconomic disparities intersect to drive adverse perinatal outcomes.

It is also important to note that smoking remains the most important modifiable risk factor associated with adverse pregnancy outcomes in our population. Following the implementation of focused smoking interventions within the Saving Babies’ Lives Care Bundle [2], including routine carbon monoxide screening in pregnancy, it will be very important to measure the impact of these interventions on smoking rates and their association with FGR and PTB in our population.

The Saving Babies Lives Care Bundle [2] has also identified screening and detection of FGR and screening for PTB as priorities for maternity services, given the strong association between both outcomes with perinatal morbidity and mortality. Our maternity hospital has developed our services in line with these recommendations, runs a preterm labour clinic, performs mid-gestation uterine artery Doppler screening for preterm FGR, and has a dedicated service for the management of FGR pregnancies. These services are universally available and accessible to all women. Furthermore, we have recently changed to a non-customised birth weight centile chart [13], which does not adjust for ethnicity and, therefore, potentially increases the recognition of FGR and small for gestational age in ethnic minority groups. This change in practice was made to ensure that all women with a previous low birth weight infant are offered additional ultrasound surveillance during their pregnancy.

Given the findings of the current study, future work will need to ensure that uptake of additional services, acceptance of interventions (e.g. aspirin prophylaxis, preterm labour interventions), and FGR detection rates are comparable amongst our different ethnic and deprivation groups. It is possible that interventions targeted at a community level may also help to improve access to specialist services and compliance with prophylactic measures. In addition, given the very significant association between FGR, PTB and lifelong cardiometabolic health risk for the offspring of affected pregnancies [15, 16], which is often compounded by genetic cardiometabolic risk, there needs to be more focus on the consequences of FGR and PTB in specific populations. Promotion of breastfeeding, monitoring of weight gain in early childhood and education regarding lifestyle interventions all have the potential to improve the long-term health of children born small and at early gestations. Within the current model of postnatal care in the UK, there are several missed opportunities to identify women and children at significant risk of poor cardiometabolic health; recognition of high-risk groups and the development of targeted, culturally sensitive interventions are urgently needed.

### Strengths and Limitations

Manchester is an ethnically diverse city in the UK, and we were therefore able to investigate the impact of several specific ethnic groups, although not all of our groups were sufficiently sized to enable the exclusion of modest effects on PTB and FGR. In contrast, our sample did not represent a wide range of deprivation deciles, and therefore, the impact of deprivation (in comparison to other studies) may have been underestimated. Importantly, the deprivation status is reflective of our population and therefore, the practical implications of the study are relevant to Manchester but may not be generalisable to other populations. Other strengths include the duration of data collection and that there was also a low level of missingness in our dataset.

Although ethnicity was self-reported by women, it is not possible to be certain that the ethnicity captured within the healthcare record accurately reflects a woman’s ethnicity. In addition, ethnicity has significant limitations in terms of capturing the complex interaction between genetic, cultural and social influences on health outcomes [17]. The accuracy of using the index of deprivation generated from booking postcodes is also a limitation. Only area deprivation derived from the booking postcode was available, which will be inaccurate in situations where a woman moves during her pregnancy. Moreover, socioeconomic status will also vary within postcode wards, and therefore, the index of deprivation is only a modest proxy for individual measures of socioeconomic deprivation. It is certainly possible that some women may be living in the most deprived areas but have a high level of income and education.

Our study was also not able to include a woman’s first language or need for interpretation as this information was not consistently reported in the health records. Health literacy access to planned and unplanned care is a potentially important determinant of health outcomes and should be included in future studies investigating health inequalities. We chose to include pregnancies in the analysis where the gestation at birth was beyond 22 weeks, which may have introduced some survivorship bias into the analysis. Furthermore, prior pregnancy complications were not consistently available and therefore not included either. Lastly, although we had a modest sample size, this was an opportunistic sample, and a formal sample size calculation was not performed. Due to the sample size limitations, the analysis was restricted to pregnancy outcomes with higher prevalence rates; more severe outcomes, including perinatal death and extreme prematurity, would require significantly larger sample sizes and were therefore not possible in this study.

## Conclusion

Although we have free and accessible health care in the UK, there remain disparities associated with ethnicity and deprivation, which influence perinatal outcomes. The reasons for this gap are likely multifactorial, encompassing lifestyle factors such as diet and physical activity, level of education, biological factors, differences in health beliefs, and level of ease in accessing healthcare services such as transport and childcare needs. Future efforts to reduce perinatal mortality and preterm birth need to consider and investigate the reasons behind these disparities if we are to make a meaningful difference in pregnancy outcomes and subsequently child health. Unless targeted interventions are implemented to combat these health inequalities, women in ethnic minority groups and those living in the most deprived areas will continue to be disproportionately affected by adverse pregnancy outcomes.

## Supplementary Information

Below is the link to the electronic supplementary material.

ESM 1 (DOCX 76.7 KB)

## Data Availability

Routinely recorded health record data is not available for sharing.
