# Supplementary material for: Examining the Magnitude of Maternal Ethnic and Socioeconomic Inequalities on Foetal Growth Restriction and Preterm Birth: A Cohort Study Set in North West England
Source: J Racial Ethn Health Disparities. 2025 Apr 16;13(4):2535–44. doi: 10.1007/s40615-025-02437-2 (PMC13346120; doi:10.1007/s40615-025-02437-2)
Supplement: Supplementary file 1 — (DOCX 76.7 KB) [file 40615_2025_2437_MOESM1_ESM.docx]

**Supplementary Information for:**

**Examining the magnitude of maternal ethnic and socioeconomic inequalities on fetal growth restriction and preterm birth; a cohort study set in North West England**

Omowunmi Omole^1^, Victoria Palin^1,^ Kylie Watson^1,2^, Jenny Myers^1^

^1^Maternal and Fetal Research Centre, Division of Developmental Biology and Medicine, the University of Manchester, St Marys Hospital, Oxford Road, Manchester, M13 9WL, UK

^2^St Mary’s Hospital, Manchester Foundation Trust, Manchester M13, 9W, UK

**Supplementary Table 1: Association between maternal characteristics and FGR (defined according to delphi definition)**

| Independent Variables (FGR) | Unadjusted OR (95% CI)  n=48,930 (N=40,120) | Adjusted OR (95% CI) for ethnicity and deprivation | Adjusted OR for ethnicity, deprivation, smoking, and maternal factors  N=48,619 (N=39,874) |
| --- | --- | --- | --- |
| Ethnicity |  |  |  |
| White British/Irish | Reference | Reference | Reference |
| Black Caribbean | **2.45 (1.82 - 3.29)** | **2.31 (1.72 - 3.11)** | **2.69 (2.01 - 3.61)** |
| Black African | **1.46 (1.26 - 1.69)** | **1.32 (1.14 - 1.54)** | **1.92 (1.64 - 2.25)** |
| Black Mixed | **1.51 (1.12 - 2.04)** | **1.43 (1.06 - 1.94)** | **1.40 (1.04 - 1.89)** |
| Pakistani | **2.30 (2.01 - 2.63)** | **2.19 (1.91 - 2.50)** | **3.10 (2.69 - 3.57)** |
| Indian | **3.03 (2.41 - 3.82)** | **2.98 (2.37 - 3.76)** | **3.58 (2.84 - 4.50)** |
| Bangladeshi | **2.98 (2.26 - 3.94)** | **2.76 (2.09 - 3.65)** | **3.81 (2.88 - 5.04)** |
| Other Asian | 1.20 (0.95 - 1.52) | 1.13 (0.89 - 1.44) | **1.52 (1.20 - 1.93)** |
| Chinese | 0.94 (0.65 - 1.36) | 0.96 (0.66 - 1.38) | 1.01 (0.70 - 1.45) |
| Other Black | 1.30 (0.81 - 2.09) | 1.24 (0.77 - 1.99) | **1.59 (0.99 - 2.53)** |
| Other White | **0.77 (0.65 - 0.92**) | **0.76 (0.64 - 0.91)** | **0.75 (0.63 - 0.89)** |
| Asian Mixed | **1.85 (1.09 - 3.16)** | **1.84 (1.08 - 3.14)** | **1.91 (1.13 - 3.22)** |
| Other | **1.23 (1.02 - 1.49)** | 1.19 (0.99 - 1.44) | **1.44 (1.19 - 1.73)** |
| Deprivation group |  |  |  |
| Middle deprivation | **1.40 (1.25 - 1.56)** | **1.24 (1.11 - 1.39)** | **1.19 (1.06 - 1.33)** |
| Most deprived | **1.43 (1.28 - 1.59)** | **1.32 (1.18 - 1.47)** | **1.24 (1.11 - 1.39)** |
| Smoker |  |  | **2.73 (2.41 - 3.09)** |
| Maternal age (years) |  |  | 1.00 (0.99 - 1.01) |
| BMI |  |  | **0.95 (0.94 - 0.96)** |
| Multiparous |  |  | **0.52 (0.48 - 0.57)** |
| Chronic hypertension |  |  | **1.99 (1.61 - 2.45)** |
| Pre-gestational diabetes |  |  | **0.55 (0.42 - 0.71)** |

**Supplementary Table 2: Association between maternal characteristics and preterm birth (before 37 weeks)**

|  | **Unadjusted OR (95% CI)**  N=48,930 (N=40,120) | **Adjusted OR (95% CI) for ethnicity and deprivation**  N=48,930 (N=40,120) | **Adjusted OR for ethnicity, deprivation, smoking, and maternal factors**  N=48,619 (N=39,874) |
| --- | --- | --- | --- |
| **Ethnicity** |  |  |  |
| White British/Irish | Reference | Reference | Reference |
| Black Caribbean | 1.16 (0.82 - 1.63) | 1.08 (0.76 - 1.53) | 1.09 (0.78 - 1.52) |
| Black African | 0.88 (0.75 - 1.04) | 0.80 (0.68 - 0.95) | 0.85 (0.72 - 1.01) |
| Black Mixed | 0.84 (0.60 - 1.19) | 0.80 (0.56 - 1.13) | 0.82 (0.58 - 1.14) |
| Pakistani | 0.89 (0.77 - 1.04) | 0.85 (0.73 - 0.99) | 0.91 (0.79 - 1.06) |
| Indian | 0.84 (0.63 - 1.13) | 0.84 (0.63 - 1.13) | 0.93 (0.70 - 1.24) |
| Bangladeshi | 0.81 (0.57 - 1.16) | 0.73 (0.51 - 1.06) | 0.78 (0.55 - 1.11) |
| Other Asian | **0.42 (0.31 - 0.58)** | **0.41 (0.30 - 0.56)** | **0.45 (0.34 - 0.62)** |
| Chinese | **0.66 (0.45 - 0.99)** | **0.66 (0.44 - 0.99)** | 0.73 (0.49 - 1.09) |
| Other Black | 0.69 (0.40 - 1.20) | 0.65 (0.37 - 1.13) | 0.72 (0.42 - 1.23) |
| Other White | **0.59 (0.49 - 0.72)** | **0.58 (0.48 - 0.70)** | **0.61 (0.51 - 0.74)** |
| Asian Mixed | 0.60 (0.30 - 1.21) | 0.60 (0.30 - 1.21) | 0.61 (0.31 - 1.20) |
| Other | **0.70 (0.57 - 0.87)** | **0.67 (0.54 - 0.83)** | **0.75 (0.61 - 0.92)** |
| **Deprivation group** |  |  |  |
| Middle deprivation | **1.22 (1.08 - 1.38)** | **1.29 (1.14 - 1.45)** | **1.24 (1.10 - 1.40)** |
| Most deprived | **1.30 (1.16 - 1.46)** | **1.38 (1.23 - 1.56)** | **1.29 (1.15 - 1.46)** |
| Smoker |  |  | **1.73 (1.52 - 1.96)** |
| Maternal age (years) |  |  | **1.01 (1.01 - 1.02)** |
| BMI |  |  | 1.00 (0.99 - 1.01) |
| Multiparous |  |  | **0.82 (0.74 - 0.91)** |
| Chronic hypertension |  |  | **3.26 (2.68 - 3.96)** |
| Pre-gestational diabetes |  |  | **2.65 (2.17 - 3.23)** |

**Supplementary Table 3. Association between maternal characteristics and FGR with birth before 37 weeks**

|  | **Prevalence (95% CI)** | **Adjusted OR for ethnicity, deprivation, smoking, and maternal factors** |
| --- | --- | --- |
| **White British/Irish** | 1.30% (1.16% - 1.45%) | Reference |
| **Black Caribbean** | 2.23% (1.43% - 3.47%) | **1.83 (1.01 - 3.30)** |
| **Black African** | 1.59% (1.29% - 1.97%) | **1.43 (1.04 - 1.96)** |
| **Black Mixed** | 1.60% (0.98% - 2.60%) | 1.21 (0.66 - 2.24) |
| **Pakistani** | 1.92% (1.61% - 2.28%) | **1.94 (1.47 - 2.57)** |
| **Indian** | 1.93% (1.33% - 2.80%) | **2.04 (1.25 - 3.34)** |
| **Bangladeshi** | 2.05% (1.31% - 3.18%) | **1.91 (1.05 - 3.47)** |
| **Other Asian** | 0.96% (0.61% - 1.52%) | 0.86 (0.49 - 1.51) |
| **Chinese** | 0.61% (0.25% - 1.45%) | 0.56 (0.21 - 1.49) |
| **Other Black** | 0.74% (0.24% - 2.27%) | 0.59 (0.17 - 2.12) |
| **Other White** | 0.91% (0.67% - 1.24%) | 0.70 (0.48 - 1.02) |
| **Asian Mixed** | 1.08% (0.35% - 3.30%) | 0.84 (0.22 - 3.18) |
| **Other** | 1.36% (1.01% - 1.84%) | 1.31 (0.89 - 1.93) |
| **Least deprived** | 1.13% (0.98% - 1.30%) | Reference |
| **Middle deprivation** | 1.39% (1.21% - 1.59%) | 1.11 (0.87 - 1.40) |
| **Most deprived** | 1.64% (1.46% - 1.83%) | **1.30 (1.03 - 1.64)** |
| **Smoker** |  | **2.19 (1.72 - 2.80)** |
| **Multiparous** |  | **0.66 (0.55 - 0.80)** |
| **Chronic hypertension** |  | **4.25 (3.04 - 5.94)** |
| **Pre-gestational diabetes** |  | 0.79 (0.49 - 1.27) |

**Supplementary Table 4: Association between maternal characteristics and spontaneous preterm birth before 37 weeks**

|  | **Unadjusted prevalence (95% CI)** | **Adjusted OR for ethnicity, deprivation, smoking, and maternal factors** |
| --- | --- | --- |
| White British/Irish | 6.19% (5.72% - 6.69%) | Reference |
| Black Caribbean | 5.67% (3.79% - 8.38%) | 0.84 (0.48 - 1.48) |
| Black African | 4.41% (3.64% - 5.34%) | **0.64 (0.47 - 0.86)** |
| Black Mixed | 4.38% (2.84% - 6.69%) | 0.57 (0.31 - 1.05) |
| Pakistani | 4.57% (3.86% - 5.40%) | **0.70 (0.54 - 0.92)** |
| Indian | 4.91% (3.47% - 6.89%) | 0.78 (0.48 - 1.28) |
| Bangladeshi | 5.14% (3.41% - 7.68%) | 0.76 (0.43 - 1.36) |
| Other Asian | 2.87% (1.95% - 4.21%) | **0.38 (0.23 - 0.65)** |
| Chinese | 4.02% (2.58% - 6.21%) | 0.55 (0.29 - 1.03) |
| Other Black | 4.32% (2.18% - 8.41%) | 0.69 (0.28 - 1.71) |
| Other White | 4.38% (3.61% - 5.32%) | **0.65 (0.49 - 0.87)** |
| Asian Mixed | 3.05% (1.15% - 7.85%) | 0.43 (0.12 - 1.48) |
| Other | 4.84% (3.84% - 6.09%) | **0.70 (0.49 - 0.99)** |
| **Least deprived** | 4.74% (4.27% - 5.25%) | Reference |
| Middle deprivation | 5.51% (4.99% - 6.08%) | **1.35 (1.10 - 1.66)** |
| Most deprived | 5.40% (4.92% - 5.93%) | **1.36 (1.10 - 1.67)** |
| Smoker |  | **2.10 (1.68 - 2.62)** |
| Maternal age (years) |  | **1.04 (1.03 - 1.06)** |
| BMI |  | **1.02 (1.01 - 1.04)** |
| Multiparous |  | **0.69 (0.58 - 0.82)** |
| Chronic hypertension |  | **1.96 (1.23 - 3.12)** |
| Pre-gestational diabetes |  | **3.60 (2.37 - 5.46)** |

Figure 1: Histogram showing IMD decile 1-10

**Supplementary Table 5 Maternal characteristics and pregnancy outcomes stratified by ethnic group**

|  | **Total** | **White British/Irish** | **Black Caribbean** | **Black African** | **Black mixed** | **Pakistani** | **Indian** | **Bangladeshi** | **Other Asian** | **Chinese** | **Other Black** | **Other White** | **Asian Mixed** | **Other** |
| --- | --- | --- | --- | --- | --- | --- | --- | --- | --- | --- | --- | --- | --- | --- |
|  | N=48,930 | N=22,432 | N=842 | N=5,198 | N=996 | N=6,408 | N=1,374 | N=923 | N=1,837 | N=800 | N=399 | N=4,416 | N=272 | N=3,033 |
| **Deprivation group** |  |  |  |  |  |  |  |  |  |  |  |  |  |  |
| Least deprived | 16,325 (33.4%) | 10,027 (44.7%) | 184 (21.9%) | 542 (10.4%) | 229 (23.0%) | 1,483 (23.1%) | 590 (42.9%) | 127 (13.8%) | 449 (24.4%) | 298 (37.2%) | 63 (15.8%) | 1,433 (32.5%) | 92 (33.8%) | 808 (26.6%) |
| Middle deprivation | 15,064 (30.8%) | 6,068 (27.1%) | 248 (29.5%) | 1,342 (25.8%) | 284 (28.5%) | 2,761 (43.1%) | 509 (37.0%) | 366 (39.7%) | 652 (35.5%) | 263 (32.9%) | 103 (25.8%) | 1,381 (31.3%) | 88 (32.4%) | 999 (32.9%) |
| Most deprived | 17,541 (35.8%) | 6,337 (28.2%) | 410 (48.7%) | 3,314 (63.8%) | 483 (48.5%) | 2,164 (33.8%) | 275 (20.0%) | 430 (46.6%) | 736 (40.1%) | 239 (29.9%) | 233 (58.4%) | 1,602 (36.3%) | 92 (33.8%) | 1,226 (40.4%) |
| **Maternal Age** | 31.1 (27.0-34.9) | 30.9 (26.5-34.8) | 30.9 (26.4-34.9) | 31.7 (27.8-35.8) | 29.6 (24.6-34.1) | 30.8 (27.3-34.6) | 31.7 (28.5-34.7) | 30.8 (26.9-34.1) | 31.2 (27.4-35.0) | 32.8 (29.8-35.6) | 30.6 (26.6-34.4) | 31.7 (27.6-35.4) | 30.0 (26.0-34.0) | 30.7 (26.8-34.6) |
| **Body Mass Index** | 25.3 (22.2-29.4) | 25.1 (22.1-29.5) | 26.4 (23.0-31.6) | 26.8 (23.4-30.8) | 25.7 (22.3-29.9) | 26.2 (23.0-29.9) | 24.6 (22.0-28.2) | 25.6 (22.6-29.2) | 25.4 (22.6-29.1) | 21.9 (20.0-24.5) | 26.3 (22.8-30.5) | 24.0 (21.6-27.6) | 24.4 (21.2-28.1) | 25.2 (22.3-28.9) |
| **Multiparous** | 28,984 (59.2%) | 12,550 (55.9%) | 533 (63.3%) | 3,628 (69.8%) | 597 (59.9%) | 4,268 (66.6%) | 745 (54.2%) | 599 (64.9%) | 1,204 (65.5%) | 391 (48.9%) | 253 (63.4%) | 2,278 (51.6%) | 146 (53.7%) | 1,792 (59.1%) |
| Missing | 56 (0.1%) | 30 (0.1%) | 0 (0.0%) | 8 (0.2%) | 0 (0.0%) | 3 (0.0%) | 0 (0.0%) | 0 (0.0%) | 3 (0.2%) | 0 (0.0%) | 0 (0.0%) | 4 (0.1%) | 0 (0.0%) | 8 (0.3%) |
| **Smoker** | 6,759 (13.8%) | 4,740 (21.1%) | 170 (20.2%) | 156 (3.0%) | 280 (28.1%) | 208 (3.2%) | 39 (2.8%) | 36 (3.9%) | 59 (3.2%) | 21 (2.6%) | 40 (10.0%) | 714 (16.2%) | 41 (15.1%) | 255 (8.4%) |
| Missing | 196 (0.4%) | 105 (0.5%) | 1 (0.1%) | 24 (0.5%) | 3 (0.3%) | 19 (0.3%) | 4 (0.3%) | 1 (0.1%) | 5 (0.3%) | 1 (0.1%) | 1 (0.3%) | 12 (0.3%) | 1 (0.4%) | 19 (0.6%) |
| **Gestation at Booking (w)** |  |  |  |  |  |  |  |  |  |  |  |  |  |  |
| Less than 10 | 13,817 (28.2%) | 6,724 (30.0%) | 262 (31.1%) | 1,061 (20.4%) | 280 (28.1%) | 2,035 (31.8%) | 482 (35.1%) | 267 (28.9%) | 457 (24.9%) | 162 (20.2%) | 101 (25.3%) | 1,094 (24.8%) | 72 (26.5%) | 820 (27.0%) |
| 10-13 | 23,111 (47.2%) | 11,284 (50.3%) | 395 (46.9%) | 2,208 (42.5%) | 493 (49.5%) | 2,827 (44.1%) | 628 (45.7%) | 450 (48.8%) | 818 (44.5%) | 410 (51.2%) | 169 (42.4%) | 2,075 (47.0%) | 138 (50.7%) | 1,216 (40.1%) |
| 13-20 | 7,073 (14.5%) | 2,535 (11.3%) | 114 (13.5%) | 1,232 (23.7%) | 160 (16.1%) | 921 (14.4%) | 161 (11.7%) | 139 (15.1%) | 306 (16.7%) | 129 (16.1%) | 77 (19.3%) | 737 (16.7%) | 35 (12.9%) | 527 (17.4%) |
| More than 20 | 4,889 (10.0%) | 1,864 (8.3%) | 71 (8.4%) | 695 (13.4%) | 62 (6.2%) | 622 (9.7%) | 102 (7.4%) | 67 (7.3%) | 255 (13.9%) | 97 (12.1%) | 52 (13.0%) | 508 (11.5%) | 27 (9.9%) | 467 (15.4%) |
| Missing | 40 (0.1%) | 25 (0.1%) | 0 (0.0%) | 2 (0.0%) | 1 (0.1%) | 3 (0.0%) | 1 (0.1%) | 0 (0.0%) | 1 (0.1%) | 2 (0.2%) | 0 (0.0%) | 2 (0.0%) | 0 (0.0%) | 3 (0.1%) |
| **Diabetes at booking** |  |  |  |  |  |  |  |  |  |  |  |  |  |  |
| Prev GDM | 1,425 (2.9%) | 302 (1.3%) | 19 (2.3%) | 209 (4.0%) | 18 (1.8%) | 458 (7.1%) | 50 (3.6%) | 65 (7.0%) | 89 (4.8%) | 32 (4.0%) | 10 (2.5%) | 62 (1.4%) | 7 (2.6%) | 104 (3.4%) |
| Type1 | 225 (0.5%) | 170 (0.8%) | 5 (0.6%) | 3 (0.1%) | 2 (0.2%) | 24 (0.4%) | 1 (0.1%) | 0 (0.0%) | 0 (0.0%) | 0 (0.0%) | 0 (0.0%) | 10 (0.2%) | 0 (0.0%) | 10 (0.3%) |
| Type2 | 330 (0.7%) | 64 (0.3%) | 7 (0.8%) | 59 (1.1%) | 7 (0.7%) | 85 (1.3%) | 11 (0.8%) | 32 (3.5%) | 17 (0.9%) | 1 (0.1%) | 3 (0.8%) | 18 (0.4%) | 7 (2.6%) | 19 (0.6%) |
| Missing | 196 (0.4%) | 112 (0.5%) | 1 (0.1%) | 18 (0.3%) | 3 (0.3%) | 19 (0.3%) | 4 (0.3%) | 1 (0.1%) | 5 (0.3%) | 1 (0.1%) | 1 (0.3%) | 10 (0.2%) | 1 (0.4%) | 20 (0.7%) |
| Hypertension at booking | 1,782 (3.6%) | 780 (3.5%) | 52 (6.2%) | 315 (6.1%) | 37 (3.7%) | 199 (3.1%) | 36 (2.6%) | 44 (4.8%) | 62 (3.4%) | 8 (1.0%) | 21 (5.3%) | 140 (3.2%) | 15 (5.5%) | 73 (2.4%) |
| Missing | 193 (0.4%) | 111 (0.5%) | 1 (0.1%) | 18 (0.3%) | 3 (0.3%) | 18 (0.3%) | 3 (0.2%) | 1 (0.1%) | 5 (0.3%) | 1 (0.1%) | 1 (0.3%) | 10 (0.2%) | 1 (0.4%) | 20 (0.7%) |
| **Perinatal death** |  |  |  |  |  |  |  |  |  |  |  |  |  |  |
| Neonatal death | 37 (0.08%) | 11 (0.05%) | 1 (0.12%) | 6 (0.12%) | 0 (0.00%) | 10 (0.16%) | 0 (0.00%) | 0 (0.00%) | 2 (0.11%) | 0 (0.00%) | 0 (0.00%) | 4 (0.09%) | 0 (0.00%) | 3 (0.10%) |
| Stillbirth | 171 (0.35%) | 71 (0.32%) | 6 (0.71%) | 23 (0.44%) | 6 (0.60%) | 19 (0.30%) | 9 (0.66%) | 4 (0.43%) | 4 (0.22%) | 2 (0.25%) | 3 (0.75%) | 10 (0.23%) | 1 (0.37%) | 13 (0.43%) |
| **Birthweight** | 3320 (2984-3645) | 3375 (3020-3700) | 3181 (2860-3462) | 3324 (3000-3645) | 3287 (2964-3634) | 3200 (2880-3512) | 3117 (2810-3420) | 3144 (2860-3420) | 3300 (2990-3600) | 3320 (3020-3606) | 3300 (2960-3660) | 3390 (3069-3700) | 3202 (2907-3470) | 3330 (3006-3650) |
| **Gestation at birth (days)** | 277 (270-284) | 276 (269-284) | 276 (269-283) | 278 (271-285) | 277 (269-284) | 276 (270-282) | 275 (270-282) | 275 (270-281) | 277 (272-283) | 278 (272-283) | 278 (270-285) | 278 (272-284) | 276 (269-283) | 278 (272-284) |
| **WHO centile** | 31.0 (12.0-57.0) | 36.0 (15.0-63.0) | 21.0 (8.0-48.0) | 29.0 (11.0-55.0) | 28.0 (11.0-54.0) | 23.0 (8.0-48.0) | 17.0 (6.0-38.0) | 19.0 (7.0-44.0) | 28.0 (11.0-56.0) | 30.0 (13.0-52.0) | 27.0 (10.0-51.0) | 34.0 (14.0-60.0) | 22.0 (10.5-44.0) | 29.0 (12.0-55.0) |
| **Centile group** |  |  |  |  |  |  |  |  |  |  |  |  |  |  |
| <3rd | 3,685 (7.5%) | 1,344 (6.0%) | 98 (11.6%) | 425 (8.2%) | 83 (8.3%) | 717 (11.2%) | 184 (13.4%) | 125 (13.5%) | 138 (7.5%) | 50 (6.2%) | 31 (7.8%) | 239 (5.4%) | 27 (9.9%) | 224 (7.4%) |
| 3^rd^-<10th | 6,568 (13.4%) | 2,514 (11.2%) | 145 (17.2%) | 742 (14.3%) | 148 (14.9%) | 1,083 (16.9%) | 295 (21.5%) | 174 (18.9%) | 281 (15.3%) | 106 (13.2%) | 59 (14.8%) | 544 (12.3%) | 44 (16.2%) | 433 (14.3%) |
| 10^th^-<90^th^ | 35,682 (72.9%) | 16,871 (75.2%) | 564 (67.0%) | 3,752 (72.2%) | 712 (71.5%) | 4,333 (67.6%) | 866 (63.0%) | 594 (64.4%) | 1,326 (72.2%) | 608 (76.0%) | 287 (71.9%) | 3,350 (75.9%) | 189 (69.5%) | 2,230 (73.5%) |
| 90^th^-<97th | 1,847 (3.8%) | 1,054 (4.7%) | 25 (3.0%) | 168 (3.2%) | 37 (3.7%) | 154 (2.4%) | 18 (1.3%) | 21 (2.3%) | 58 (3.2%) | 24 (3.0%) | 14 (3.5%) | 184 (4.2%) | 8 (2.9%) | 82 (2.7%) |
| ≥97th | 1,148 (2.3%) | 649 (2.9%) | 10 (1.2%) | 111 (2.1%) | 16 (1.6%) | 121 (1.9%) | 11 (0.8%) | 9 (1.0%) | 34 (1.9%) | 12 (1.5%) | 8 (2.0%) | 99 (2.2%) | 4 (1.5%) | 64 (2.1%) |
| **FGR** | 4,137 (8.5%) | 1,570 (7.0%) | 109 (12.9%) | 469 (9.0%) | 95 (9.5%) | 790 (12.3%) | 201 (14.6%) | 134 (14.5%) | 145 (7.9%) | 55 (6.9%) | 34 (8.5%) | 259 (5.9%) | 30 (11.0%) | 246 (8.1%) |
| **FGR (birth <37 weeks)** | 680 (1.4%) | 288 (1.3%) | 18 (2.1%) | 82 (1.6%) | 16 (1.6%) | 122 (1.9%) | 26 (1.9%) | 18 (2.0%) | 17 (0.9%) | 5 (0.6%) | 3 (0.8%) | 40 (0.9%) | 3 (1.1%) | 42 (1.4%) |
| **PTB (<37 weeks)** | 3,400 (6.9%) | 1,720 (7.7%) | 72 (8.6%) | 361 (6.9%) | 68 (6.8%) | 447 (7.0%) | 93 (6.8%) | 62 (6.7%) | 75 (4.1%) | 47 (5.9%) | 25 (6.3%) | 234 (5.3%) | 15 (5.5%) | 181 (6.0%) |
| **PTB (<34 weeks)** | 1,045 (2.1%) | 509 (2.3%) | 31 (3.7%) | 134 (2.6%) | 22 (2.2%) | 142 (2.2%) | 29 (2.1%) | 16 (1.7%) | 22 (1.2%) | 13 (1.6%) | 7 (1.8%) | 54 (1.2%) | 5 (1.8%) | 61 (2.0%) |
| **Labour onset** |  |  |  |  |  |  |  |  |  |  |  |  |  |  |
| Induced | 14,423 (29.5%) | 7,008 (31.2%) | 242 (28.7%) | 1,443 (27.8%) | 327 (32.8%) | 1,858 (29.0%) | 350 (25.5%) | 246 (26.7%) | 495 (26.9%) | 192 (24.0%) | 107 (26.8%) | 1,202 (27.2%) | 87 (32.0%) | 866 (28.6%) |
| No labour | 12,840 (26.2%) | 5,938 (26.5%) | 198 (23.5%) | 1,526 (29.4%) | 214 (21.5%) | 1,700 (26.5%) | 404 (29.4%) | 251 (27.2%) | 490 (26.7%) | 147 (18.4%) | 111 (27.8%) | 1,033 (23.4%) | 58 (21.3%) | 770 (25.4%) |
| Spontaneous | 21,666 (44.3%) | 9,486 (42.3%) | 402 (47.7%) | 2,229 (42.9%) | 455 (45.7%) | 2,850 (44.5%) | 620 (45.1%) | 426 (46.2%) | 852 (46.4%) | 461 (57.6%) | 181 (45.4%) | 2,180 (49.4%) | 127 (46.7%) | 1,397 (46.1%) |
| Missing | 1 (0.0%) | 0 (0.0%) | 0 (0.0%) | 0 (0.0%) | 0 (0.0%) | 0 (0.0%) | 0 (0.0%) | 0 (0.0%) | 0 (0.0%) | 0 (0.0%) | 0 (0.0%) | 1 (0.0%) | 0 (0.0%) | 0 (0.0%) |
| **Method of Birth** |  |  |  |  |  |  |  |  |  |  |  |  |  |  |
| Forceps | 5,264 (10.8%) | 2,620 (11.7%) | 44 (5.2%) | 308 (5.9%) | 77 (7.7%) | 675 (10.5%) | 171 (12.4%) | 84 (9.1%) | 193 (10.5%) | 95 (11.9%) | 28 (7.0%) | 608 (13.8%) | 25 (9.2%) | 336 (11.1%) |
| Ventouse | 2,035 (4.2%) | 940 (4.2%) | 18 (2.1%) | 143 (2.8%) | 26 (2.6%) | 269 (4.2%) | 57 (4.1%) | 45 (4.9%) | 84 (4.6%) | 71 (8.9%) | 10 (2.5%) | 212 (4.8%) | 18 (6.6%) | 142 (4.7%) |
| Elective caesarean | 8,019 (16.4%) | 3,961 (17.7%) | 104 (12.4%) | 820 (15.8%) | 125 (12.6%) | 995 (15.5%) | 250 (18.2%) | 138 (15.0%) | 315 (17.1%) | 92 (11.5%) | 64 (16.0%) | 673 (15.2%) | 30 (11.0%) | 452 (14.9%) |
| Emergency caesarean | 6,730 (13.8%) | 2,695 (12.0%) | 130 (15.4%) | 1,021 (19.6%) | 135 (13.6%) | 927 (14.5%) | 233 (17.0%) | 183 (19.8%) | 219 (11.9%) | 93 (11.6%) | 65 (16.3%) | 552 (12.5%) | 38 (14.0%) | 439 (14.5%) |
| Spontaneous vaginal | 26,874 (54.9%) | 12,214 (54.4%) | 546 (64.8%) | 2,905 (55.9%) | 633 (63.6%) | 3,539 (55.2%) | 663 (48.3%) | 473 (51.2%) | 1,026 (55.9%) | 448 (56.0%) | 232 (58.1%) | 2,371 (53.7%) | 161 (59.2%) | 1,663 (54.8%) |
| Missing | 8 (0.0%) | 2 (0.0%) | 0 (0.0%) | 1 (0.0%) | 0 (0.0%) | 3 (0.0%) | 0 (0.0%) | 0 (0.0%) | 0 (0.0%) | 1 (0.1%) | 0 (0.0%) | 0 (0.0%) | 0 (0.0%) | 1 (0.0%) |
| **Post partum haemorrhage (PPH)** |  |  |  |  |  |  |  |  |  |  |  |  |  |  |
| Minor PPH | 9,210 (18.8%) | 4,209 (18.8%) | 130 (15.4%) | 973 (18.7%) | 179 (18.0%) | 1,171 (18.3%) | 310 (22.6%) | 150 (16.3%) | 367 (20.0%) | 208 (26.0%) | 81 (20.3%) | 837 (19.0%) | 47 (17.3%) | 548 (18.1%) |
| Major PPH | 2,840 (5.8%) | 1,198 (5.3%) | 65 (7.7%) | 387 (7.4%) | 39 (3.9%) | 341 (5.3%) | 74 (5.4%) | 48 (5.2%) | 108 (5.9%) | 77 (9.6%) | 22 (5.5%) | 285 (6.5%) | 12 (4.4%) | 184 (6.1%) |
| Missing | 426 (0.9%) | 188 (0.8%) | 6 (0.7%) | 56 (1.1%) | 4 (0.4%) | 67 (1.0%) | 9 (0.7%) | 11 (1.2%) | 15 (0.8%) | 6 (0.8%) | 2 (0.5%) | 34 (0.8%) | 5 (1.8%) | 23 (0.8%) |
